# The association of loneliness after sudden bereavement with risk of suicide attempt: a nationwide survey of bereaved adults

**DOI:** 10.1007/s00127-020-01921-w

**Published:** 2020-07-18

**Authors:** Alexandra L. Pitman, Michael B. King, Louise Marston, David P. J. Osborn

**Affiliations:** 1 UCL Division of Psychiatry, University College London, Maple House, 149 Tottenham Court Road, London, W1T 7NF UK; 2 Camden and Islington NHS Foundation Trust, St Pancras Hospital, 4 Saint Pancras Way, London, NW1 0PE UK; 3 UCL Research Department of Primary Care and Population Health, University College London, Gower St, London, WC1E 6BT UK

**Keywords:** Loneliness, Social isolation, Suicide attempt, Suicidal ideation, Stigma, Bereavement

## Abstract

**Purpose:**

We aimed to test the hypothesis that among people who experience sudden bereavement, loneliness is associated with post-bereavement suicide attempt and post-bereavement suicidal ideation, even when adjusting for network size.

**Methods:**

We analysed cross-sectional data collected in the 2010 UCL Bereavement Study, to identify 3193 respondents who had experienced sudden bereavement. We used multivariable logistic regression to test for an association between loneliness (using a newly-developed eight-item loneliness measure) and post-bereavement suicide attempt and suicidal ideation, adjusting for socio-demographic factors, pre-bereavement depression and self-harm, and network size.

**Results:**

Among bereaved adults, loneliness was significantly associated with probability of post-bereavement suicide attempt (AOR 1.19; 95% CI 1.14–1.25) and of post-bereavement suicidal ideation (AOR 1.24; 95% CI 1.20–1.28), with estimates unchanged by adding perceived stigma of the bereavement to adjusted models. There was no association between suicide bereavement and loneliness (adjusted coefficient 0.22; 95% CI − 0.12 to 0.45; *p* = 0.063). The association of loneliness and suicide attempt risk was similar whether participants were bereaved by suicide or not.

**Conclusions:**

People who report feeling lonely after sudden bereavement are more likely to make a suicide attempt after their loss, even when taking into account their network size and the perceived stigma of the sudden bereavement. There is no evidence that the effects of loneliness on suicidality are specific to suicide bereavement. This work identifies loneliness as a potential target for suicide prevention interventions among bereaved people. It also fuels interest in longitudinal research investigating loneliness as a putative mediator of suicide risk.

**Electronic supplementary material:**

The online version of this article (10.1007/s00127-020-01921-w) contains supplementary material, which is available to authorized users.

## Introduction

The World Health Organisation (WHO) reports that 800,000 people die by suicide annually worldwide, and that for each of those deaths approximately 20 other people attempt suicide [1]. Amongst long-established risk factors such as mental illness and alcohol misuse, the WHO also hypothesises that a sense of isolation may be a risk factor for suicidality [1]. Whilst social isolation is defined objectively as a lack of social contacts, the concept of loneliness has been defined as the unpleasant experience that occurs when a person’s network of social relationships is deficient either quantitatively or qualitatively [2]; the subjective, unwelcome feeling of a lack or loss of companionship [2]; and as an inner, subjective sense of not being sufficiently socially connected [3]. Loneliness and social isolation are both distinct from the concept of social support; the emotional, tangible (practical), informational, and companionship support arising from either formal (professional) or informal (friends and relatives) sources [4]. Both social isolation and loneliness have recently become the focus of health and social policy research, and the adverse physical and mental health effects of loneliness are becoming increasingly well recognised [5], including the association between loneliness or social isolation and suicidality [6]. This is a particular concern amongst young people, in whom loneliness is prevalent and also stigmatising [5]. The hypothesised links between loneliness and suicidality are that lonely individuals feel dissatisfied with life [7], and that their perceptions of limited social connections contribute to a desire to die [8]. Other mechanisms suggested include the contribution of loneliness to chronic stress, the link with cognitive factors such as low self-esteem or an external locus of control, and the mediating role of depression [6]. Theoretical models of suicidality consider the role of *thwarted belongingness;* a construct describing what arises when the fundamental need to form and maintain strong, stable interpersonal relationships is unmet, resulting in feelings of disconnection [8, 9]. The Interpersonal Theory of Suicide considers thwarted belongingness as comprising (a) loneliness and (b) the absence of reciprocal caring relationships [8], and confirmatory factor analysis shows convergent associations of thwarted belongingness with loneliness [10]. The Integrated Motivational–Volitional (IMV) model of suicide proposes that factors such as thwarted belongingness and perceived burdensomeness (the view that one’s existence is a burden on friends, family and/or society) govern the transition from suicidal ideation to suicide attempt [11]. Studies conducted to date have found loneliness to be associated with suicidal behaviour in Canadian [12] and English [6] general population samples, even when adjusting for common mental disorder [6], and in specific populations limited to war veterans [13, 14], adolescents [15], and sexual minority groups [16]. However, no studies have investigated this in a bereaved population.

The loneliness arising from sudden bereavement is a topic worthy of further investigation because sudden bereavement is itself a risk factor for suicide [17], particularly where the death was due to suicide [18, 19]. Although the mediators of suicide risk after sudden bereavement remain unidentified, factors such as loneliness, social isolation, and poor social support are implicated [20]. Loneliness after sudden loss may arise from the abrupt loss of a confiding relationship or companionship. The taboo around sudden or violent losses can also engender social isolation due to others’ awkwardness and avoidance [21]. The perceived stigma of sudden bereavement, defined as the subjective awareness of others’ negative attitudes towards the loss [22], is likely to be a major contributor to loneliness after loss. This stigma arises not only from others’ avoidance but also from a perception of them gossiping about the death or casting blame [23]. Although stigma is perceived after all sudden deaths, most commonly due to embarrassment or a fear of appearing socially incompetent, it is particularly pronounced after deaths by suicide and other unnatural causes [24, 25]. This is likely to be due to the long history of religious and cultural sanctions against suicide, stigmatising the act and those mourning it [26], and others’ views about irresponsibility in the case of accidental death [21]. The perceived stigma of sudden bereavement is associated with risk of suicidal thoughts and suicide attempts [22], and there is also evidence that perceived stigma contributes to suicide attempt risk after suicide bereavement [25].

In addition to suicidality, unexpected bereavement is also associated with a range of incident psychiatric disorders [27], and it is possible that these might lead to or compound loneliness. Not all individuals will be adversely affected, and grief is a non-pathological process. However, risk of poor outcomes is likely to be governed by factors such as personal vulnerability [28], the quality of attachment to the person who died [29], and the degree to which social support buffers the negative impact of a traumatic life event [30]. Further work is needed to describe the relationships between loneliness, social isolation and suicidality after a sudden loss to further our understanding of the roles that social isolation and loneliness might play in the pathway to suicidality. No previous studies have measured the association between loneliness after the sudden loss of a close friend or relative and suicide-related outcomes. We aimed to investigate whether greater loneliness was associated with a higher probability of suicide attempt and suicidal ideation after sudden bereavement in a population-based sample of adults in the United Kingdom. We also aimed to test the hypothesis that associations differ by mode of bereavement (suicide bereavement versus non-suicide bereavement), such that the magnitude of the association would be greater in the suicide-bereaved, and that the main association might partly be accounted for by the perceived stigma of the bereavement.

## Methods and materials

### Study design

We analysed cross-sectional data collected in the UCL Bereavement Study: a 2010 survey of all staff and students aged 18–40 at 37 UK higher educational institutions (HEIs) who had experienced the sudden bereavement of a close friend or relative since reaching the age of 10. Full details of sampling for this closed online survey have been described elsewhere [22], including the survey instrument [25]. The focus was on young adults given policy concerns about their risk of suicide [31] and their tendency not to engage with services when in distress [32–34]. Sampling via global email lists avoided the biases associated with recruiting a help-seeking sample, and was felt to be the most efficient, comprehensive and pragmatic means of recruiting a hard-to-reach population of young adults [35].

Of 5085 respondents to the survey, we included those who: consented to participate; completed the loneliness item of the Social Functioning Questionnaire (SFQ) and all seven items within a scale measuring perceptions of social support received from family and friends; and specified their mode of bereavement (*n* = 3193). This sample included respondents who had experienced sudden bereavement due to sudden natural causes (*n* = 1952), sudden unnatural causes (*n* = 666), or suicide (*n* = 575).

### Ethical approval

Ethical approval for analysing data from the UCL Bereavement Study was obtained from the UCL Research Ethics Committee in 2010 (reference number: 1975/002).

### Measures

#### Exposure: loneliness

Our exposure was loneliness using eight items chosen to represent the construct of loneliness, as used in the seven-yearly Adult Psychiatric Morbidity Surveys (APMS), a regular representative population survey in England [36]. One item was taken from the SFQ [37]; an eight-item self-report scale used to measure current social function. Seven items were taken from a scale measuring perceptions of social support received from family and friends, used in the 1987 Health and Lifestyle survey in England [38]. These capture respondents’ sense of whether relatives and friends can: be relied upon; make them feel loved, valued and accepted; and support and encourage them. The items therefore convey the degree to which one’s relationships create a perception of social belonging and acceptance as well as of emotional connectedness, and companionship. By reverse scoring them in this loneliness measure, higher scores captured the perception of lacking belongingness, acceptance, connectedness and companionship in one’s social relationships.

The development of this continuous measure, scored from 0 to 17, is described in “Appendix”.

#### Outcome: suicide attempt

Our outcomes were: self-reported suicide attempt (“Have you ever made an attempt to take your life, by taking an overdose of tablets or in some other way?”) [39] and self-reported suicidal ideation (“Have you ever thought of taking your life, even though you would not actually do it?”) [40] post-bereavement. These standardised measures were again taken from the APMS [36], qualified by whether each was before or after the sudden bereavement, or both, to derive an incident measure.

#### Covariates

We also measured the following covariates:

- socio-economic status, using the UK Office for National Statistics Standard Occupational Classification [41];
- depression, using the Composite International Diagnostic Interview (CIDI) screen for lifetime depression [42], qualified by whether the onset of these core symptoms of low mood and anhedonia occurred before or after the sudden bereavement, to derive an incident measure
- possible personality disorder, using the 8-item self-report Standardised Assessment of Personality-Abbreviated Scale (SAPAS), a standardised instrument with demonstrated reliability and validity in screening for likely personality disorder in psychiatric out-patient samples [43, 44]
- perceived stigma of the sudden loss, measured using the stigmatisation sub-scale of the validated Grief Experience Questionnaire (GEQ) [26]
- network size, using a continuous measure of primary group size derived from the Interview Measure for Social Relationships (IMSR); a standardised instrument with demonstrated reliability [45], as used in the APMS [36] with waves of population norms [46]. This captures the numbers of friends and relatives (aged 16 years and over) respondents felt close to, sub-categorised into numbers of close relatives, extended family members, and friends.

We selected nine potential confounding variables on the basis of existing literature and clinical judgement: age, gender, socio-economic status, pre-bereavement depression, pre-bereavement (suicidal and non-suicidal) self-harm; and primary group size.

Missing data for model covariates and outcomes varied from < 1% (for age, gender, group size, pre-bereavement depression, pre-bereavement self-harm, pre-bereavement suicide attempt, and pre-bereavement suicidal thoughts) to 3% (for socio-economic status).

### Statistical analysis

We described median values (and interquartile ranges) or means (and standard deviations) for our exposure variable and each descriptive variable, and reported *p* values for tests of univariate associations with each using linear regression. Our threshold for significance was set at *p* < 0.05.

We investigated the relationship between loneliness and outcomes using multilevel regression models with HEI as random effect, to take into account the clustering effect at the HEI level. We used multivariable logistic regression to investigate the relationship between loneliness (continuous measure) and two binary outcomes (post-bereavement suicide attempt, post-bereavement suicidal ideation), adjusting for socio-demographic factors, pre-bereavement psychopathology, and primary group size, as described above.

To test whether the effect of loneliness on outcomes varied by mode of bereavement (suicide versus non-suicide loss), we added this binary variable as an interaction term to adjusted models, using a less stringent *p* value threshold (*p* < 0.1) to reflect the limited statistical power of interaction tests. We also used multivariable logistic regression to investigate whether suicide bereavement is associated with post-bereavement loneliness, adjusting for the same potential confounders as in the list above.

Finally, we tested whether adding the perceived stigma of bereavement to our main adjusted models attenuated the association between loneliness and suicide-related outcomes, to provide evidence supporting a partially mediating effect. To set this in context, we also described the association between loneliness and perceived stigma.

Models were fitted using complete case analysis. We ran a priori defined sensitivity analyses to assess the robustness of our main findings when taking into account biases introduced by 3% missing data on socio-economic status, using best-case and worst-case scenarios to impute missing values.

All analyses were conducted using Stata version 13 (StataCorp. 2013. *Stata Statistical Software: Release 13*. College Station, TX: StataCorp LP).

## Results

### Participant characteristics

The majority of our sample of 3193 adults were female (81%), of white ethnicity (90%), bereaved by sudden natural causes (61%), and reporting the death of a relative (71%) (Table 1). The sample was relatively evenly split between participants aged 18–21 years (41%) and those aged 22–40 years (59%). Mean age of respondents was 25·1 years (standard deviation [SD] 6·3, whilst mean age at bereavement was 20.1 years (SD 6.04; median 19; interquartile range [IQR] 16–23). Mean time elapsed since bereavement was 5 years (SD 5·3; range 1 day–30 years). The age of the deceased varied from 0 (for miscarriage or stillbirth) to 100 years, with a median age of 47 years (IQR 23–63). In the total sample mean loneliness scores were 2.49 (SD 2.70; median 1; IQR 1–3). Median network size in this population was 11 (IQR 7–16; mean 12.9; SD 9.1). The proportion scoring positive for possible personality disorder was 37%, ranging from 22% reporting “difficulty making and keeping friends” and 22% describing themselves as a “loner”, to 63% reporting being “a perfectionist” and 72% as “a worrier”.

**Table 1:** Univariate associations of loneliness scores (continuous variable) with participant characteristics (*n* = 3193)

	Proportion of overall sample *n* (%)	Loneliness score median (IQR)	*p* value^a^
*Socio-demographic characteristics*			
Gender			
Male	592 (19)	2 (1,4)	
Female	2600 (81)	1 (1, 3)	** < 0·001**
Missing	1 (< 1)		
Age of participant (years)			
Aged 18–21	1276 (40)	1 (1, 3)	0**·**065
Aged 22–40	1917 (60)	1 (1, 3)	
Relationship status			
Single	2238 (70)	1.5 (1, 4)	** < 0·001**
Within a relationship	950 (30)	1 (1, 3)	
Missing	5 (< 1)		
Self-defined ethnicity			
White	2875 (90)	1 (1, 3)	** < 0·001**
Non-white	315 (3)	2 (1, 4)	
Missing	3 (< 1)		
Socio-economic status^b^			
Social classes 1.1 and 1.2	1972 (62)	1 (1, 3)	0**·**179
Social classes 3–7 and 9	1126 (35)	2 (1, 4)	
Missing	95 (3)		
Educational status			
Attained maximum A level equivalent	1389 (44)	1 (1, 3)	0.072
Attained degree level or above	1797 (56)	1 (1, 3)	
Missing	7 (< 1)		
*Clinical characteristics*			
Post-bereavement suicidal thoughts			
Yes	1512 (47)	2 (1, 5)	** < 0·001**
No	1662 (52)	1 ( 1, 2)	
Missing	19 (< 1)		
Post-bereavement suicide attempts			
Yes	209 (7)	3 (1, 7)	** < 0·001**
No	2958 (93)	1 (1, 3)	
Missing	26 (< 1)		
Pre-bereavement depression^c^			
Yes	629 (20)	1 (1, 5)	** < 0·001**
No	2559 (80)	1 (1, 3)	
Missing	5 (< 1)		
Pre-bereavement (suicidal and non-suicidal) self-harm			
Yes	714 (22)	2 (1, 4)	** < 0·001**
No	2448 (77)	1 (1, 3)	
Missing	31 (1)		
Personality disorder screen positive ^d^			
Yes	1182 (37)	2 (1, 5)	** < 0·001**
No	2011 (63)	1 (1, 2)	
Missing	0 (0)		
*Characteristics of the bereavement*			
Kinship to the deceased			
Blood-related	2276 (71)	1 (1, 3)	0.530
Non blood-related	906 (28)	1 (1, 3)	
Missing	11 (< 1)		
Years since bereavement			0.390
Less than 2 years	982 (31)	1 (1, 3)	
Over 2 years	2211 (69)	1 (1, 3)	
Missing	0 (0)		
Perceived stigma of the bereavement^e^ (dichotomised at the median)			** < 0·001**
High	1525 (48)	2 (1, 5)	
Low	1667 (52)	1 (1, 2)	

^a^*p* values for univariate associations of characteristics with loneliness scores

^b^Socio-economic status using the 5 categories from UK Office for National Statistics

^c^Measured using CIDI screen for depression [42]

^d^Measured using SAPAS screen for possible personality disorder [44]

^e^Measured using stigmatisation sub-scale of the Grief Experience Questionnaire [26]

Univariate associations of loneliness with socio-demographic and clinical characteristics, and measures of social connectedness, are shown in Table 1. Loneliness scores were significantly higher in men, those who defined themselves as single or non-white, those classified as of lower socio-economic status, those reporting depression or self-harm prior to bereavement, those reporting post-bereavement suicidal thoughts or attempts, those screening positive for possible personality disorder, and those with greater perceived stigma scores.

### Association between loneliness scores and outcomes

In an adjusted analysis (Table 2), higher loneliness scores were associated with a significantly higher probability of post-bereavement suicide attempts (AOR 1.19; 95% CI 1.14–1.25) and of post-bereavement suicidal ideation (AOR 1.24; 95% CI 1.20–1.28). Stepped adjustments are reported in Supplementary Table 3.

**Table 2:** Estimates of the associations between loneliness scores and outcomes in bereaved participants (*n* = 3193)

	OR	95% CI	*p* value	AOR ^↑^	95% CI	*p* value
Outcome						
Probability of post-bereavement suicide attempt	1.22	1.17–1.27	**< 0.001**	1.19	1.14–1.25	**< 0.001**
Adding perceived stigma of bereavement to above adjusted model				1.13	1.08–1.19	**< 0.001**
Probability of post-bereavement suicide ideation	1.26	1.22–1.30	**< 0.001**	1.24	1.20–1.28	**< 0.001**
Adding perceived stigma of bereavement to above adjusted model				1.18	1.13–1.22	**< 0.001**

^**↑**^Adjusted for age, gender, socio-economic status, pre-bereavement depression, pre-bereavement (suicidal and non-suicidal) self-harm; and primary group size

There was no evidence to support an interaction of mode of bereavement with suicide attempt (*p* = 0.7211) or suicidal ideation (*p* = 0.6343). Associations with suicide attempt were similar whether someone had been bereaved by suicide (AOR 1.21; 95% CI 1.10–1.33; *p* = 0.005) or by non-suicide causes (AOR 1.19; 95% CI 1.12–1.25; *p* < 0.001). Similarly, associations with suicidal ideation were similar whether someone had been bereaved by suicide (AOR 1.26; 95% CI 1.16–1.37; *p* < 0.001) or non-suicide causes (AOR 1.23; 95% CI 1.19–1.28; *p* < 0.001). There was no significant association between suicide bereavement and loneliness (adjusted coefficient 0.22; *p* = 0.063; 95% CI − 0.12 to 0.45), when adjusted for the same nine covariates included in the main model.

There was a significant association between loneliness and perceived stigma of bereavement, in an unadjusted model [unadjusted coefficient 0.574 (95% CI 0.525–0.623; *p* ≤ 0.001) and when adjusting for the same nine covariates (adjusted coefficient 0.532 (95% CI 0.480–0.583; *p* ≤ 0.001)].

Adding scores for perceived stigma of sudden bereavement to final models (Table 2) slightly attenuated the association between loneliness and suicide attempt (AOR 1.13; 95% CI 1.08–1.19), and between loneliness and suicidal ideation (AOR 1.18; 95% CI 1.14–1.23), with both associations remaining significant.

We added a post hoc test for an interaction with stigma (dichotomised at the median into high versus low scores) and found no evidence for an interaction with suicide attempt (*p* = 0.6343) or suicidal ideation (*p* = 0.2053).

### Sensitivity analysis

In sensitivity analyses simulating potential biases introduced by 3% missing data for socio-economic status the magnitude, direction and precision of adjusted odds ratios for the association between loneliness scores and outcomes were unchanged.

## Discussion

### Main findings

The findings of this analysis of British cross-sectional data support our hypothesis that people who feel lonely after the sudden death of a friend or relative are more likely to experience suicidal thoughts and to make a suicide attempt after the loss, even when adjusting for social network size and for the perceived stigma of the bereavement. The magnitude of our risk estimates were such that the odds of making a suicide attempt or of having suicidal thoughts increased by 19% and 24%, respectively with each point increase on our loneliness measure. Potential explanations for this association include loneliness contributing to substance misuse, to a sense that no-one would understand their problems, and to the intensity of fantasies about reunion with the deceased, otherwise considered a normal grief reaction. The associations remained significant after adding stigma to final models, and we found no interaction with high versus low stigma score. This is surprising because of the observed association between the stigma of sudden bereavement and suicidal ideation and attempt [22]. This suggests that a sense of loneliness is independently associated with probability of suicidality among people who experience sudden bereavement.

Our interaction tests found that feeling lonely had no greater effect on suicidality among people bereaved by suicide than in people bereaved by non-suicide deaths, despite findings from the same sample identifying the suicide-bereaved as having a significantly higher probability of post-bereavement suicide attempt [25]. This again is surprising, and suggests that for the suicide-bereaved other factors relating to suicide loss make additional contributions to their suicide risk. It is plausible that the stigma of suicide may condition the suicide-bereaved to normalise loneliness as an expected consequence of suicide loss. Instead, other factors influencing suicide risk are likely to include psychiatric disorder [18, 19], substance misuse [47], and a reluctance to seek help for mental health problems [48]. Risk of mood disorders and post-traumatic stress disorder are elevated in people bereaved by suicide [19]. People who have experienced suicide bereavement describe binge drinking or drug taking as a coping strategy in the immediate aftermath of the death [47]. They are also less likely to consult their general practitioner than bereaved controls, despite their greater probably of mental illness [19], which may be due to the stigma of suicide loss reducing motivation to seek help [49–53]. Self-stigma is certainly an important factor in dissuading people from seeking help for mental health problems, primarily due to reluctance to disclose a mental health condition [54]. Suicide bereavement is characterised not only by stigma, but also by a sense of responsibility, rejection, and shame when compared to bereaved controls [55]. A more detailed exploration of patterns of help-seeking for suicidality among the suicide-bereaved in relation to shame and stigma may be warranted, as it is possible that a reluctance to seek help accounts for their greater risk of suicide attempt. Finally, it is possible that carer burden prior to suicide loss might give rise to higher levels of pre-bereavement loneliness [56], although this requires specific testing.

In our sample there was a high overall proportion of people with post-bereavement suicidal ideation (47%), greatly exceeding those for the maximum lifetime prevalence of suicidal ideation (20.6%) in any corresponding age group within the APMS 2007 representative sample of the English population [36]. The proportion of those reporting a suicide attempt (7%) in our sample, during a period spanning a mean of 5 years since their loss, was similar to the maximum lifetime prevalence of suicide attempt (7.3%) in any corresponding age group within the same APMS 2007 representative sample [36]. Among a group in which suicidality is commonplace, screening for loneliness may be a useful means of identifying individuals at risk of suicide attempt and initiating a conversation about appropriate ways of alleviating loneliness in the context of that person’s unique circumstances.

### Results in the context of other studies

Few other studies have used nationwide population-based samples to investigate the associations of loneliness with suicide-related outcomes. Previous work in Canada found a strong association between loneliness and suicidal ideation (OR 10.5; 95% CI 8.4–13.1) and self-harm (OR 13.5; 95% CI 9.3–19.6) in a representative household sample [12]. However, that study measured loneliness using one unvalidated item capturing responses to the question “How often do you feel alone?”, rated on a 5-point scale from 0 (never) to 4 (very often), and odds ratios were unadjusted [12]. An analysis of English household survey data found that those with higher levels of loneliness were significantly more likely to report past year suicidal ideation (AOR 11.09; 95% CI 6.91–17.79) and past year suicide attempts (AOR 17.37; 95% CI 5.51–54.72) [6]. This analysis adjusted for age, sex, educational qualifications, ethnicity, marital status, wealth, employment status, alcohol dependence, social support, physical health conditions, stressful life events and common mental disorder, and the magnitude of the effect sizes were large. However, confidence intervals were wide, and the analysis was based on the single item from the SFQ, whereas ours used the SFQ item together with seven complementary items capturing perceptions of belongingness, companionship, acceptance, and connectedness.

## Strengths and limitations

We analysed data from a large, UK-wide sample of 3193 bereaved adults using a newly-validated loneliness measure with good face and content validity, and internal consistency, albeit without data validating it against an established measures of loneliness. Our hypotheses were formulated on the basis of current theory and clinical experience, and our models were adjusted for pre-selected potential confounders, including pre-bereavement psychopathology. Results were robust to sensitivity analysis simulating the biases potentially introduced by missing data. However, our use of cross-sectional data limits interpretation of the chronology of the pathways between loneliness scores (measured currently) and outcomes (measured at any time after the bereavement), and it is possible that suicidality engenders further loneliness or that the relationships are bidirectional. Our logistic regression did not capture the time between bereavement and suicidality outcome measures, nor whether respondents related their suicidality to the bereavement. As bereavement was as distal as 30 years for some participants, and a mean of 5 years, it is possible that the experience of current loneliness may relate to more recent factors, including current mental illness. It is also possible that suicidality since bereavement was explained by other unmeasured factors. Further longitudinal work is necessary to understand the directionality, and potential reinforcing effect, of these relationships, and how the strength of associations varies over time. Our sample was predominantly white, female and highly-educated, and this limits generalisability of our findings to settings outside HEIs and in other countries.

## Clinical and research implications

This study has identified loneliness after a sudden bereavement to be associated with suicidality, whether or not the loss was due to suicide. Our findings suggest a need for agencies that support bereaved people, including counsellors, general practitioners, voluntary sector organisations, and informal networks, to inquire about loneliness and consider ways of addressing this. However, the stigma of admitting to feeling lonely may preclude identification of the problem, particularly in those who wish to mask their sense of isolation. As yet the evidence base for interventions that address loneliness and prevent adverse mental health outcomes is in its infancy. However, systematic reviews of studies evaluating the effectiveness of interventions to address loneliness in people with existing mental health problems [57] and in the general population [58]. These tentatively favour interventions addressing maladaptive cognitions, such as biases in cognition towards negative aspects of the social context. Pending trial evidence in bereaved populations, referral to a peer bereavement support group may be an acceptable means of increasing connectedness among bereaved people, particularly those who feel alienated from their non-bereaved peers. Such groups may provide the only access to people who can understand them, or the only setting in which expressing grief is acceptable [59]. They may also be a setting in which selective attention towards others’ failure to offer support after loss, and other maladaptive cognitions, might be challenged. Even for those who have family and friends around them, peer support groups offer the potential for connectedness with people who have a shared understanding of the experience of bereavement, as well as the opportunity to help others [59].

Longitudinal work to explore the nature of the relationships between loneliness, stigma, social support, and suicidality, would help determine the comparative contribution of loneliness, stigma, and poor informal support as putative mediators of the association between sudden bereavement and suicide-related outcomes. Understanding these relationships would help determine whether there is a need to develop and trial individual-level or community-level anti-stigma interventions among those have been bereaved and who feel lonely and stigmatised. Investigating the relationship between loneliness and hopelessness is also warranted. Theoretical models of the interrelationship between loneliness and suicidal ideation suggest that the effects of loneliness on future suicidality operate through its influence on hopelessness [60]. However, when tested, findings support an alternative model in which hopelessness predicts both loneliness and suicidality, with no direct relationship between loneliness and suicidality beyond hopelessness [60]. Our study did not measure hopelessness, but testing these hypotheses in a bereaved population would contribute further to our understanding of these mechanisms. Finally, further investigation of the relationship between loneliness and entrapment is warranted using longitudinal approaches, as entrapment is a key factor in theoretical models of suicide [61].

## Conclusions

Our results confirm an association between feeling lonely after the sudden death of a friend or relative and an increased risk of suicidal thoughts and suicide attempt, which is not explained by a lack of friends or family or by feeling stigmatised by the loss. Our most striking finding is that suicide bereavement is not associated with feeling lonely, and that loneliness had no greater effect in people bereaved by suicide, despite their greater risk of suicide attempt. Further work using longitudinal data is needed to understand the potentially complex associations between loneliness, stigma, mental illness, social support and suicidality in people who experience bereavement. This will help identify how we might mitigate the negative health effects of sudden bereavement, as part of suicide prevention efforts.

### Electronic supplementary material

Below is the link to the electronic supplementary material.

Supplementary file1 (DOCX 13 kb)

## Appendix: Development of loneliness measure

### Items

#### Loneliness item from the Social Functioning Questionnaire (SFQ)

Participants provide fixed choice responses to the following statement, scored as:

□ “Almost all the time" □ “Much of the time" □ “Not usually" □ “Not at all"

“I feel lonely and isolated from other people".

Measure of perceived social support, from the 1987 Health and Lifestyle survey in England:

Participants provide fixed choice responses to the seven statements below, scored as:

□ not true □ partly true □ certainly true

“I would now like you to think about your family and friends. (By family I mean those who live with you as well as those elsewhere.) Here are some comments people have made about their family and their friends. For each statement, please say whether it is: not true, partly true, or certainly true for you”.

- There are people I know amongst my family and friends who do things to make me happy.
- There are people I know amongst my family and friends who make me feel loved.
- There are people I know amongst my family and friends who can be relied on no matter what happens.
- There are people I know amongst my family and friends who would see that I am taken care of if I needed to be.
- There are people I know amongst my family and friends who accept me just as I am.
- There are people I know amongst my family and friends who make me feel an important part of their lives.
- There are people I know amongst my family and friends who give me support and encouragement.

In the UCL Bereavement Study these survey questions followed a set of questions on the social network, priming respondents to think about their wider social network, including friends, relatives, and acquaintances.

### Scoring

The SFQ item is conventionally scored as: 0 (“Not at all"), 1 (“Not usually"), 2 (“Much of the time"), or 3 (“Almost all the time"), with higher scores indicating greater loneliness.

The measure of perceived social support was originally scored as: 1 (“not true”), 2 (“partly true”) or 3 (“certainly true), generating total scores between 7 and 21, and transformed into three categories corresponding to no lack of perceived social support, a moderate lack, and a severe lack [38]. Other studies have scored items from 0 to 2, generating total scores between 0 and 14, and demonstrating good internal consistency (Cronbach’s alpha 0.88) [62]. For the current study we rescored these items in reverse order to 0, 1, 2 to match the directionality of the SFQ item scoring, and reduced all scores by 1 to match the lowest scoring (0) for SFQ. This resulted in a measure scored from 0 to 17, and with higher scores at the scale’s negative pole (denoting a lack of belongingness, acceptance, connectedness and companionship).

### Tests of validity

#### Datasets

To test construct validity of this eight-item measure we used two British cross-sectional datasets: the APMS general population samples for 2007 [36] and 2014 [63], and the UCL Bereavement Study sample [25]. The Department of Health’s seven-yearly APMS national population surveys provide data on the prevalence of both treated and untreated psychiatric disorders in the English adult population aged 16 years and over. We included in our analysis of APMS data from 2007 and 2014 adults who had completed the single SFQ item on loneliness, and the seven items on perceived social support, and were aged below 65 years (*n* = 10,420). We included in our analysis of UCL Bereavement Study data those who had completed the same eight items (*n* = 3193). This sample included respondents who had experienced sudden bereavement due to sudden natural causes (*n* = 1952), sudden unnatural causes (*n* = 666), or suicide (*n* = 575).

#### Construct validity

We assessed construct validity by conducting Exploratory Factor Analysis (EFA) of the eight selected questionnaire items to ascertain which to retain. To do this we first used combined data on people aged below 65 in the APMS 2007 and 2014 household surveys. We used a more stringent eigenvalue threshold than the default value of 1 by the scree test (*screeplot* in Stata) to identify the natural bend or break point in the data curve, and used the number of data points above this break to identify the number of factors to retain [64]. We then derived a Kaiser–Meyer–Olkin (KMO) measure of sampling adequacy using the standard threshold of 0.7. We summed all eight items and tested internal consistency of this scale by measuring Cronbach’s alpha, and then retested this seven times, each time dropping one item to compare Cronbach’s alpha values.

#### Convergent validity

To test convergent validity, a sub-type of construct validity, we tested univariate associations of the 8-item loneliness measure with measures theoretically related to the construct of loneliness. We chose variables capturing social connectedness, and tested for associations with the following using the nonparametric equality-of-medians test:

- marital status;
- primary group size, using a component of the Interview Measure of Social Relations (IMSR) as used in the 1987 Health and Lifestyle survey in England [38] (categorised as 0–3 individuals/4 to 8/9 or more);
- number of encounters with primary group members in the last week, using a different component of the IMSR, (categorised as: contact with no-one in last week; contact with 1–5 people; with 6–10 people; with 11–15 people; with 16–20 people; with 21–30 people; with 31+ people);
- 8 individual items within the Standardised Assessment of Personality—Abbreviated Scale (SAPAS) screen for personality disorders (difficulty with relationships, tendency to be a loner/mistrustful/to lose one’s temper/impulsive/anxious/dependent/perfectionist).
- screen for possible personality disorder, using the SAPAS [44] score with the standard cutoff at a threshold of four traits.

We also tested for an association with number of children, pre-hypothesising no association, in keeping with previous findings of no association between loneliness and the presence, number, and age of children for married men and women [65].

We then repeated these tests using the UCL Bereavement Study dataset. Our tests of convergent validity additionally tested for an association with a variable unavailable in the APMS dataset: living situation (categorised as: alone; living with spouse/partner; single parent living with children; living with parents; living with other relatives; sharing accommodation with non-relatives; student hall of residence or student hostel; temporary hostel or bed and breakfast accommodation; homeless; other).

### Factor analysis

Our EFA of eight items using the APMS combined datasets showed that all items mapped to a one factor structure, with this factor having an eigenvalue of 3.87. In contrast, other factors’ eigenvalues ranged from − 0.15 to 0.18. Factor loadings on the one factor identified were 0.32 for the SFQ item on perceived loneliness, and between 0.61 and 0.80 for the seven items on the reverse-scored perceived social support scale. KMO values on all items were above 0.89 (exceeding the standard threshold of 0.7), with an overall value of 0.91. By convention, this confirmed that the variables had sufficient in common to warrant a factor analysis. On summing all eight items, Cronbach’s alpha was 0.80 (designated by convention as strong internal consistency), with an average inter-item covariance of 0.08. On retesting Cronbach’s alpha by removing individual items, its value never dropped below 0.76, therefore denoting acceptable internal consistency and that all eight items should be retained. Our tests of convergent validity showed that the loneliness measure was significantly associated with all variables tested apart from number of children in the household.

Repeating these tests in the UCL Bereavement Study dataset, EFA again identified a one factor structure, with this factor having an eigenvalue of 3.59. On contrast, other factors’ eigenvalues ranged from − 0.15 to 0.12. Factor loadings on the one factor identified were 0.44 for the SFQ item on perceived loneliness, and between 0.61 and 0.76 for the seven items on the reverse-scored perceived social support scale. KMO values on all items were above 0.89 (again exceeding the threshold), with an overall value of 0.91. On summing all eight items, Cronbach’s alpha was 0.84 (again designating strong internal consistency), with an average inter-item covariance of 0.10. On retesting Cronbach’s alpha by removing individual items, its value again never dropped below 0.80. Our tests of convergent validity showed that the loneliness measure was significantly associated with all variables tested apart from number of children in the household and perfectionistic traits, as pre-hypothesised. These findings established good face, content, construct, and convergent validity of this continuous measure.
