# Supplementary material for: The association of loneliness after sudden bereavement with risk of suicide attempt: a nationwide survey of bereaved adults
Source: Soc Psychiatry Psychiatr Epidemiol. 2020 Jul 18;55(8):1081–92. doi: 10.1007/s00127-020-01921-w (PMC7395013; doi:10.1007/s00127-020-01921-w)
Supplement: Supplementary file 1 — Supplementary file1 (DOCX 13 kb) [file 127_2020_1921_MOESM1_ESM.docx]

# Manuscript title: The association of loneliness after sudden bereavement with risk of suicide attempt

Supplementary table 3: **Estimates of the associations between loneliness scores and outcomes in bereaved participants (n=3,193) showing all steps in adjusted models**

|  | **OR** | **95 % CI** | **p-value^*^** |
| --- | --- | --- | --- |
| **Outcome** |  |  |  |
| **Probability of post-bereavement suicide attempt** |  |  |  |
| unadjusted | 1.22 | 1.17 – 1.27 | **<0·001** |
| adjusted for age, gender, and socio-economic status | 1.22 | 1.17 - 1.27 | **<0·001** |
| adjusted for age, gender, socio-economic status, pre-bereavement depression, and pre-bereavement (suicidal and non-suicidal) self-harm | 1.20 | 1.15 – 1.25 | **<0·001** |
| adjusted for age, gender, socio-economic status, pre-bereavement depression, pre-bereavement (suicidal and non-suicidal) self-harm, and primary group size (final model) | 1.19 | 1.14 - 1.25 | **<0·001** |
| *adjusted for all above covariates plus perceived stigma of bereavement* | 1.13 | 1.08 - 1.19 | **<0·001** |
| **Probability of post-bereavement suicide ideation** |  |  |  |
| unadjusted | 1.26 | 1.22 – 1.30 | **<0·001** |
| adjusted for age, gender, and socio-economic status | 1.26 | 1.22 – 1.30 | **<0·001** |
| adjusted for age, gender, socio-economic status, pre-bereavement depression, and pre-bereavement (suicidal and non-suicidal) self-harm | 1.25 | 1.21 – 1.29 | **<0·001** |
| adjusted for age, gender, socio-economic status, pre-bereavement depression, pre-bereavement (suicidal and non-suicidal) self-harm, and primary group size (final model) | 1.24 | 1.20 -1.28 | **<0·001** |
| *adjusted for all above covariates plus perceived stigma of bereavement* | 1.18 | 1.13 - 1.22 | **<0·001** |

**^*^** 2-sided significance threshold of p=0**·**05
